# Bioinformatics analysis identified shared differentially expressed genes as potential biomarkers for Hashimoto's thyroiditis-related papillary thyroid cancer

**DOI:** 10.7150/ijms.63402

**Published:** 2021-08-13

**Authors:** Chang Liu, Yu Pan, Qinyu Li, Yifan Zhang

**Affiliations:** 1 Department of Nuclear Medicine, Ruijin Hospital, Shanghai Jiao Tong University School of Medicine, Shanghai 200025, China.; 2 Department of General Surgery, Ruijin Hospital, Shanghai Jiao Tong University School of Medicine, Shanghai 200025, China.

**Keywords:** Papillary thyroid cancer, Gene analysis, Biomarkers, Hashimoto's thyroiditis

## Abstract

**Background:** Although the etiology of Hashimoto's thyroiditis (HT), a common autoimmune endocrine disease, is unknown, studies suggest a potential association with genetic factors and environmental conditions inducing excessive iodine intake. Additionally, HT patients have a high risk of papillary thyroid cancer (PTC), which is probably related to the chronic inflammation and autoimmune pathologic process occurring in HT, as it is thought to be associated with neoplastic transformation.

**Methods:** Bioinformatics approaches can identify differentially expressed genes (DEGs) and analyze DEG functions in diseases. R software was used in this study to identify DEGs in HT and PTC using data in Gene Expression Omnibus (GEO). The online tools DAVID, Reactome, and AmiGO were employed for annotation, visualization, and integration of DEGs related to HT and PTC, and the STRING database and Cytoscape software were applied to predict and visualize protein-protein networks (PPIs) for DEG-encoded proteins. Coexpressed DEGs in HT and PTC were validated by reverse transcription PCR (RT-PCR).

**Results:** In total, 326, 231, and 210 DEGs in HT specimens and samples of central PTC and PTC invasive areas, respectively, were detected. According to the PPI network, *PTPN6*, *HLA-A*, *C3AR1*, *LCK* and *ITGB2* are hub genes among HT-DEGs, whereas *FN1*, *CDH2*, *SERPINA1*, and *CYR61* are PTC-DEG hub genes. The shared DEGs *LTF* and *CCL21* were validated by RT-PCR. Both bioinformatics and RT-PCR analyses showed *LTF* and *CCL21* to be upregulated in HT tissues and downregulated in PTC tissues.

**Conclusions:** We identified that expression of *LTF* and *CCL21* are significantly different in HT and PTC, suggesting an underlying association between HT and PTC.

## Introduction

Hashimoto's thyroiditis (HT), also known as lymphocytic thyroiditis, is a common autoimmune endocrine disease with an increasing prevalence in recent decades 1, 2. Chronic inflammation is connected to neoplastic transformation, and it is known that patients with autoimmune diseases have increased risks for some types of tumors 3, 4, with neoplastic transformation often occurring 5-8. For example, there is evidence that HT patients have a high risk of thyroid cancer, the most common endocrine malignancy, than the general population 9-15. According to the literature, the pathogenesis of HT-related thyroid cancer may be due to the diffuse lymphocyte infiltration, apoptosis of thyroid epithelial cells, fibrous replacement, and follicular destruction in HT.

However, controversy exists regarding whether HT is related to papillary thyroid cancer (PTC), which accounts for 95% of all thyroid cancer cases and has increasing prevalence worldwide. Some studies have found that high levels of antithyroid peroxidase antibody (TPO-Ab) and increased thyroid-stimulating hormone (TSH) are related to HT-related thyroid tumors. Thyroid cancer patients with HT have higher TSH levels than those with benign nodules. Higher TSH levels have been found to be related to terminal cancer in Caucasian patients with well-differentiated thyroid cancer 16-20. Moreover, Kim et al 21 reported a very strong association between PTC and HT (odds ratio [OR] =2.96; 95% CI, 1.81-4.85; P < 0.001). On the other hand, Haymart et al 16, 22 found only borderline significance for the association between HT and PTC (P = 0.051).

Clinically, diagnosis of HT is mainly based on US-FNA, and tools for accurately predicting the risk of PTC are lacking 23. In this study, we analyzed and validated shared differentially expressed genes (DEGs) of HT and PTC, which may be used as biomarkers for HT-related PTC.

## Methods

### Gene expression data acquisition

Two expression profiling array datasets (GSE29315 and GSE6004) were downloaded from Gene Expression Omnibus (GEO) 24 and analyzed using Affymetrix Human Genome Array with the annotation platforms GPL8300 [HG_U95Av2] and GPL570 [HG-U133_Plus_2]. The GSE29315 dataset contains 6 HT samples and 8 thyroid hyperplasia (TH) samples. GSE6004 includes 7 PTC samples with a paired central PTC and invasive areas obtained from 7 patients, and 4 control thyroid tissue samples.

### Data processing

The quality of the profile data used was assessed by relative log expression (RLE) analysis through the affyPLM package of R software 25. Boxplots for GSE29315 and GSE6004 are shown in supplement Fig 1. Robust multiarray averages (RMAs) and mismatch probes (PMs) were created for processing the datasets, including background correction, normalization, expression calculation, and probe integration. The classical Bayesian method utilized by the limma package in R software was applied to analyze DEGs for three comparisons (HT vs TH, central area of PTC vs normal, and invasive area of PTC vs normal). P-values were adjusted based on the Benjamini-Hochberg method, and fold-changes (FCs) were calculated by the false discovery rate (FDR) procedure. PTC-DEGs with |log2-fold change| > 1.5 and P < 0.05 were selected. HT-DEGs were filtered according to the criteria |log2-fold change| > 2 and P < 0.05. A heat map of DEGs was constructed using the gplots package, and Venn diagrams were generated using Venny 2.1 (https://bioinfogp.cnb.csic.es/tools/venny/index.html) to identify overlapping genes.

### Annotation and functional characterization of DEGs

Gene Ontology (GO) 26 annotation of characteristic genes functions for three categories, biological process (BP), cellular component (CC), and molecular function (MF), was performed, and Kyoto Encyclopedia of Genes and Genome (KEGG) 27 was used to analyze signaling pathways. The identification of DEG functions and signaling pathways can be annotated, visualized, and integrated using several online tools, including DAVID (https://david-d.ncifcrf.gov/) 28, Reactome 29, and AmiGO. 30. In this study, we used DAVID to perform GO functional enrichment analysis for DEGs of HT and PTC, the AmiGO database to verify enrichment of GO terms, and the DAVID and Reactome databases to analyze KEGG signaling pathways, with a threshold of P < 0.05.

### Construction of protein-protein interaction (PPI) networks

The STRING database (v11.0) was used to assess protein interactions, including physical and functional interactions. The results were imported into Cytoscape software (v3.7.2) for visualization of the relationships of interacting genes 31, and interactions with a confidence score > 0.4 were considered significant.

### Association of DEGs shared between thyroid and endocrine diseases and cancer

Associations between DEGs and related diseases were analyzed by using Comparative Toxicogenomic Database (CTD). We assessed DEGs in endocrine diseases and cancers to evaluate relationships between gene products, to produce extensive networks and to predict novel associations 32.

### HT and PTC patient specimens

This study enrolled five surgically confirmed PTC patients from November 2019 to December 2019; all specimens included HT tissues. We divided the specimens into 3 groups: HT, PTC, and corresponding PTC-adjacent normal tissues, which were used as the normal control. The study design was approved by the Ethical Review Board of Ruijin Hospital, Shanghai Jiao Tong University School of Medicine. All patients signed informed consent.

### Quantitative real-time reverse transcription PCR (qRT-PCR)

RNA was extracted from tissues using TRIzol (Sangon Biotech, Shanghai, China). All RNAs were first reverse-transcribed to cDNA using PrimeScript™ RT Master Mix (TaKaRa Bio Inc., Japan) (37 °C for 15 min, 85 °C for 5 sec, and cooled to 4 °C). RT-PCR of *PC* and the reference gene *β-actin* was performed following the protocol of the TB Green® Premix Ex Taq™ II kit (TaKaRa Bio Inc., Japan) and an Applied Biosystems 7500 Real-Time PCR System. The temperature cycling protocol consisted of 30 sec denaturation at 95 °C, followed by 40 cycles of 95 °C for 5 sec and 60 °C for 34 sec; the 40 cycles were followed by 95 °C for 15 sec, 60°C for 1 min, and 95 °C for 15 sec. The primers used in this study are provided in Supplementary Materials Table S2. The gene expression data were normalized to those of *β-actin*. In this study, cycle threshold (Ct) values below 35 were included. The 2^-ΔΔCt^ method was applied to evaluate gene expression levels.

## Results

### Identification of DEGs in two GEO datasets

There were 67,302 probes corresponding to 8,575 and 20,461 genes identified in the GSE29315 and GSE6004 datasets, respectively. We confirmed 231 and 210 DEGs from the central and invasive areas of PTC specimens by comparing with control thyroid tissue samples, respectively. A total of 326 DEGs were found in HT compared with thyroid hyperplasia samples.

### Functional analysis of HT- and PTC-DEGs

**Figure 1:**
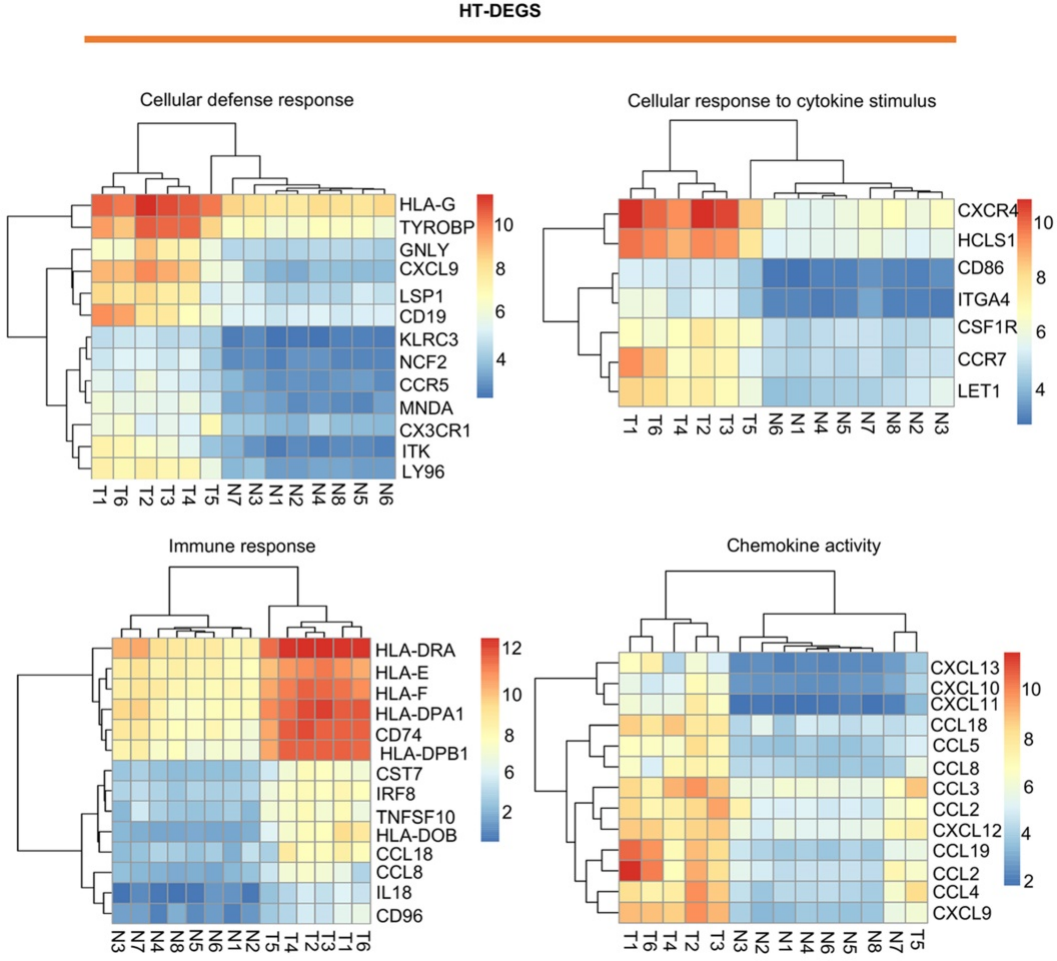
Visualization of HT-related DEGs with a heatmap: DEGs related to the terms cellular defense response, cellular response to cytokine stimulus, immune response, and chemokine activity. T indicates HT tissues, and N indicates thyroid hyperplasia tissues. Red indicates high expression, and blue indicates low expression.

**Figure 2:**
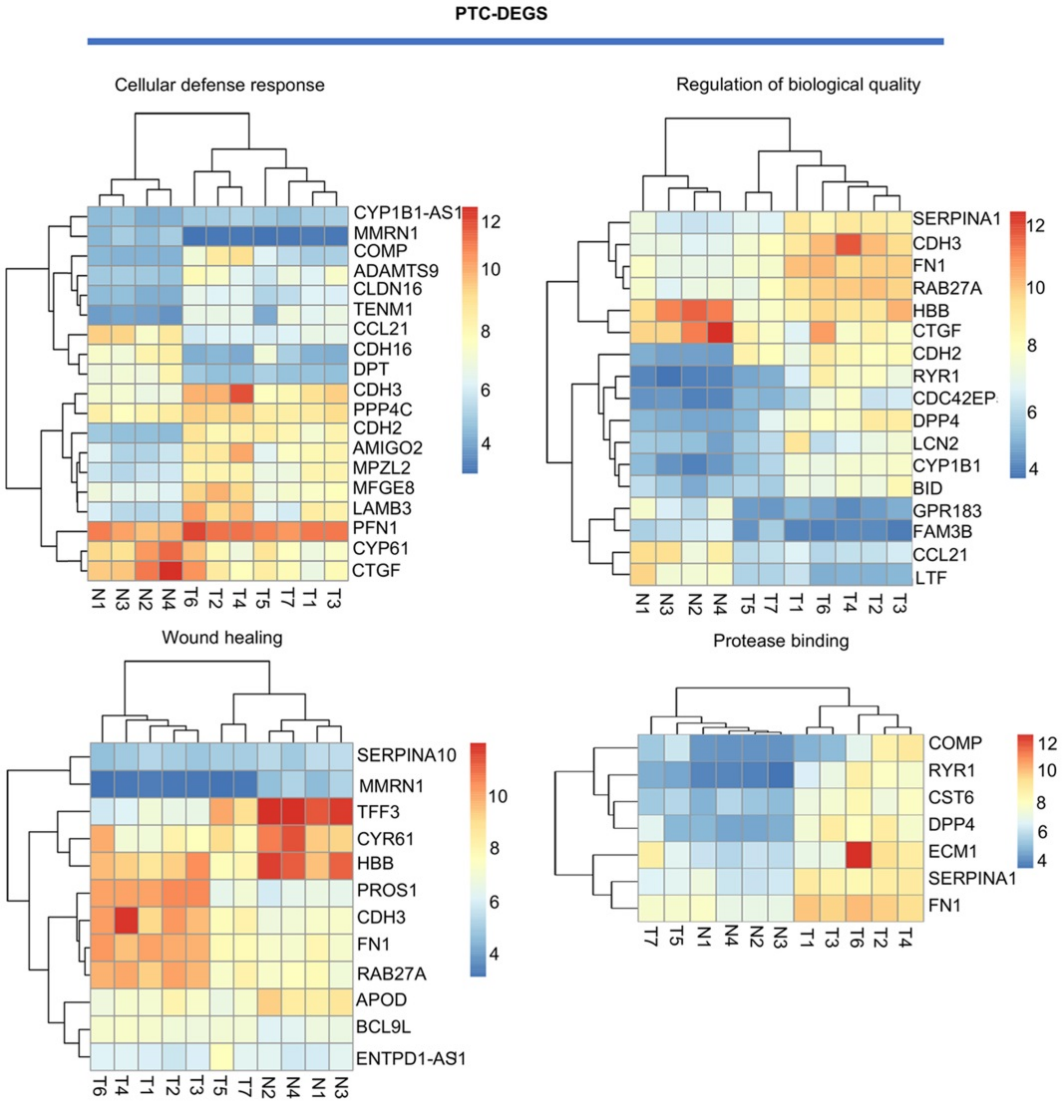
Visualization of PTC-related DEGs with a heatmap: DEGs related to the terms cell adhesion, wound healing, protease binding, and regulation of biological quality. T indicates PTC tissues, and N indicates normal thyroid tissues. Red indicates high expression, and blue indicates low expression.

**Figure 3:**
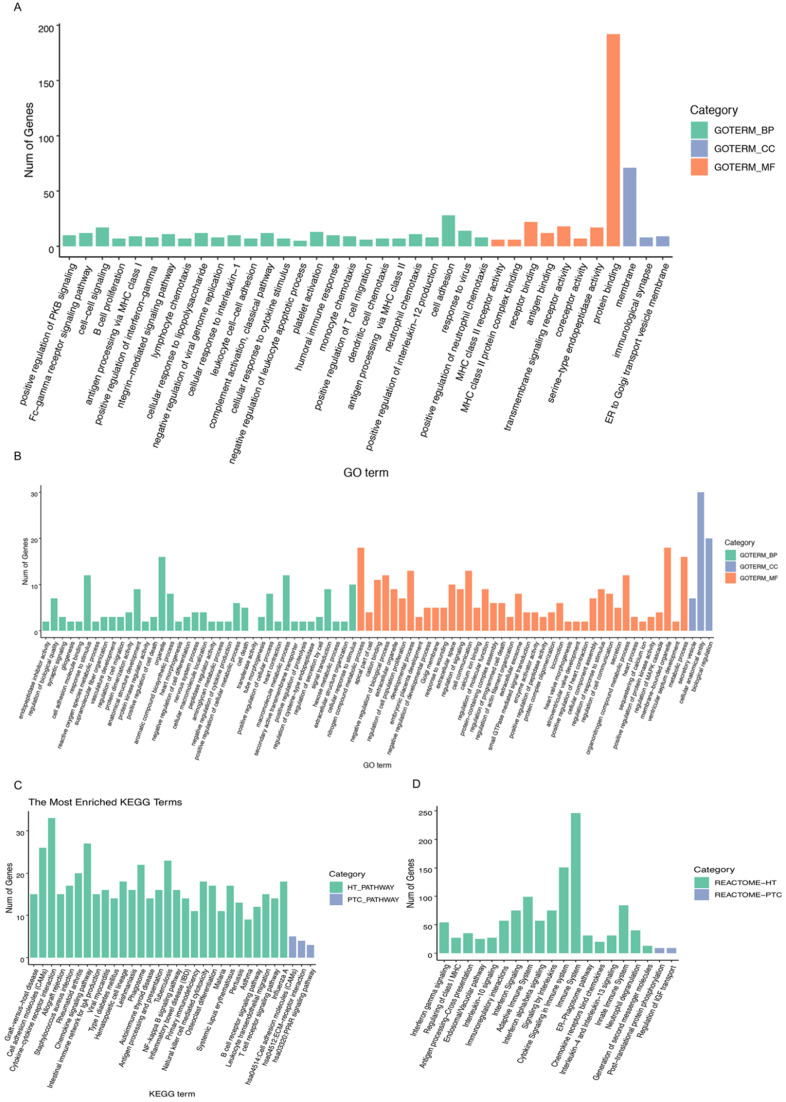
Biological process and pathway analysis: **A, B** GO categories related to HT-DEGs and PTC-DEGs. Green indicates biological process, blue indicates cellular component, and orange indicates molecular function. **C, D** KEGG and Reactome pathway analyses of DEGs shared between HT and PTC. Green indicates HT-related pathways, and blue indicates PTC-related pathways.

A heatmap of HT-related DEGs related to the immune response and cancer signaling is depicted in Fig. 1. In addition, Fig. 2 shows PTC-DEGs associated with cancer signaling, energy metabolism, and gene regulation. Based on DAVID analysis, the most enriched biological processes of HT-DEGs were the immune response (fold enrichment: 9.11; P value: 1.15E-47), inflammation (fold enrichment: 6.04; P value: 7.97E-21), signaling pathways mediated by chemokines (fold enrichment: 13.51; P value: 1.12E-14), type I interferon signaling (fold enrichment: 14.16; P value: 3.32E-14), and B cell receptor signaling (fold enrichment: 15.79; P value: 3.84E-14). In terms of cellular components, the external side of the plasma membrane (fold enrichment: 9.14; P value: 5.28E-22) and extracellular space (fold enrichment: 2.64; P value: 3.57E-12) were identified. Enriched molecular functions were found to be mainly related to chemokine activity (fold enrichment: 14.97; P value: 3.78E-11), peptide antigen binding (fold enrichment: 18.15; P value: 1.96E-08) and transmembrane signaling receptor activity (fold enrichment: 4.74; P value: 2.65E-07) (Fig. 3A).

For PTC-DEGS, the biological process terms enriched were mainly related to the regulation of cell-cell adhesion (fold enrichment: 4.03; P value: 1.99E-05), the regulation of neuropeptides (fold enrichment: 7.33; P value: 0.001), blood coagulation (fold enrichment: 4.69; P value: 0.003), and gene expression (fold enrichment: 3.77; P value: 0.005). The PTC-DEGs also showed enrichment of the terms extracellular region (fold enrichment: 2.71; P value: 2.54E-07) and proteinaceous extracellular matrix (fold enrichment: 5.91; P value: 5.85E-06) for the cellular component category. With respect to molecular function, the terms protease binding (fold enrichment: 10.78; P value: 8.96E-06), heparin binding (fold enrichment: 7.66; P value: 2.24E-05), and calcium ion binding (fold enrichment: 2.66; P value: 0.002) were identified as enriched (Fig. 3B). Subsequently, the AmiGO database was used to verify GO term enrichment, as indicated in Supplementary Materials Table S1.

The results of KEGG pathway analysis are provided in Fig. 3C, suggesting HT-DEG-related pathways to be mainly associated with graft-versus-host disease (fold enrichment: 16.54; P value: 4.15E-14), cell adhesion molecules (CAMs) (fold enrichment: 6.66; P value: 4.66E-14), cytokine-cytokine receptor interactions (fold enrichment: 4.94; P value: 5.08E-14), the intestinal immune network for IgA production (fold enrichment: 11.61; P value: 1.24E-11), and the NF-κB signaling pathway (fold enrichment: 6.69; P value: 9.52E-09). KEGG terms regulation of cellular adhesion (fold enrichment: 4.75; P value: 0.019) and interaction of extracellular matrix-related receptors (fold enrichment: 6.20; P value: 0.025) were enriched for PTC-DEGs. Function and pathway enrichment analyses via the Reactome database were used to identify additional relationships, which are shown in Fig. 3D.

### PPI networks of HT-DEGs and PTC-DEGs

**Figure 4:**
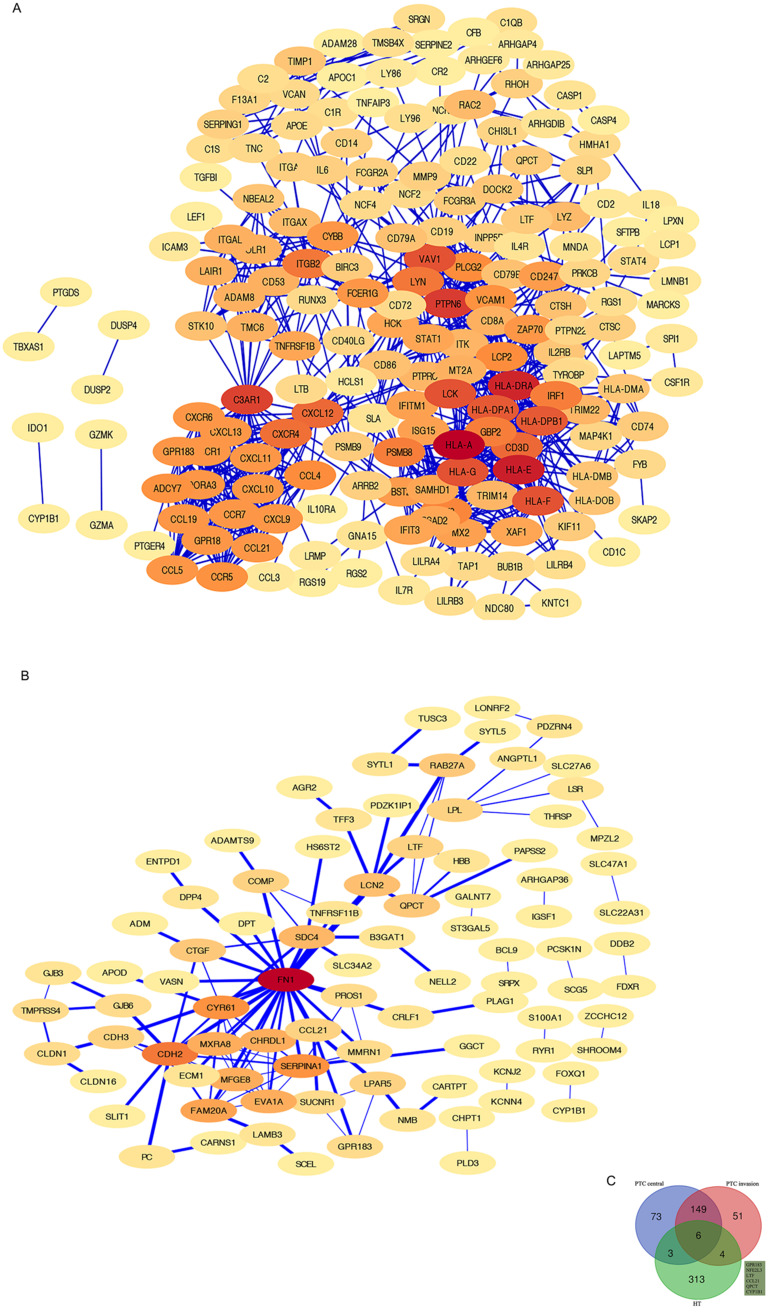
Shared DEG analysis with PPI networks and Venn diagrams: A, B PPI networks of HT- and PTC-related DEGs (threshold > 0.4). Red indicates hub genes. C Shared DEGs related to HT and the center and invasive areas of PTC. Shared genes, including *GPR183, NFE2 L3, LTF, CCL21, QPCT,* and* CYP1B1*, are shown.

The HT-DEGs and PTC-DEGs were further imported into the online database STRING, respectively. Interacting genes were examined after removing genes without interaction. A total of 184 nodes were included in the PPI network of HT-DEGs and 89 nodes in the PPI network of PTC-DEGs. The PPI network was visualized in Cytoscape software, as illustrated in Fig. 4. Gene scoring identified significant nodes related to HT, including protein tyrosine phosphatase nonreceptor 6 (*PTPN6*; degree = 31), human leukocyte antigen A (*HLA-A*; degree = 39), C3a anaphylatoxin chemotactic receptor (*C3AR1*; degree = 30), tyrosine protein kinase Lck (*LCK*; degree = 28), and integrin beta-2 (*ITGB2*; degree = 23) (Fig. 4A). Hub genes fibronectin (*FN1*; degree = 22), cadherin (*CDH2*; degree = 13), alpha-1-antitrypsin (*SERPINA1*; degree = 11), and cysteine-rich 61 (*CYR61*; degree = 11) were identified in the PTC-DEG PPI network (Fig. 4B).

**Figure 5:**
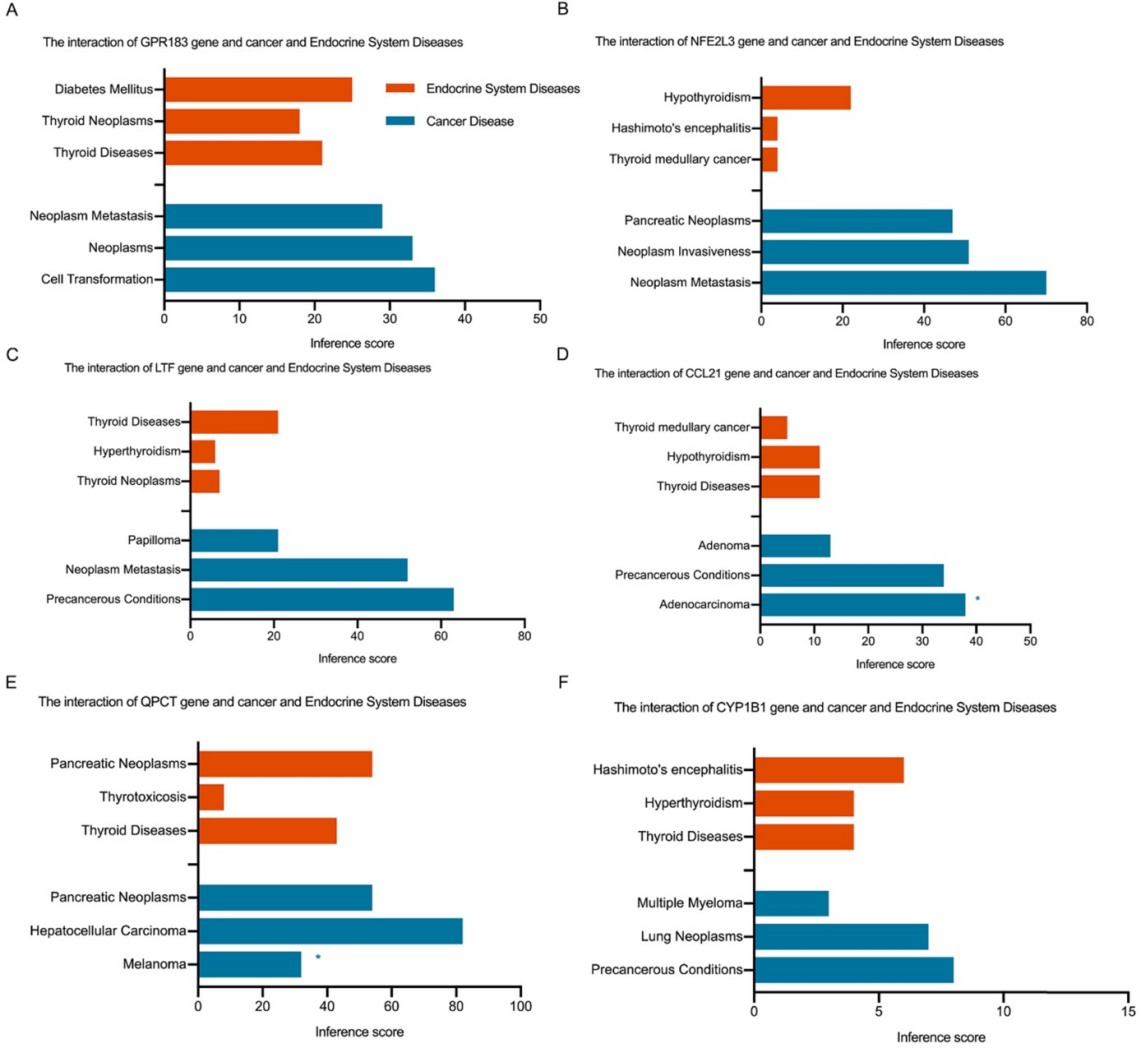
Interaction of shared DEGs, endocrine system diseases, and cancer diseases identified via the CTD database. Orange indicates shared DEGs related to endocrine system diseases, and blue indicates shared DEGs related to cancer diseases. * indicates proven evidence in this disease.

Fig. 4C shows the Venn diagram used to identify shared DEGs between the 2 groups (HT and PTC). Notably, a total of six shared DEGs (G-protein coupled receptor 183 (*GPR183*, log FC is 2.51 in HT vs -2.29 in PTC), NRF2-related factor 3 (*NFE2L3*, log FC is 2.00 in HT vs 1.91 in PTC), lactotransferrin (*LTF*, log FC is 2.59 in HT vs -2.71 in PTC), C-C motif chemokine 21 (*CCL21*, log FC is 3.65 in HT vs -2.58 in PTC), glutamine-peptide cyclotransferase-like protein (*QPCT*, log FC is 2.32 in HT vs 2.63 in PTC), and cytochrome P450 1B1 (*CYP1B1*, log FC is 2.89 in HT vs 2.43 in PTC)) were identified. The CTD database was used to analyze the targets of the shared DEG-associated diseases, and the results are provided in Fig. 5. Overall, the results suggest that DEGs shared between HT and PTC correlate with cancer and thyroid diseases.

### Validation of shared DEGs

**Figure 6:**
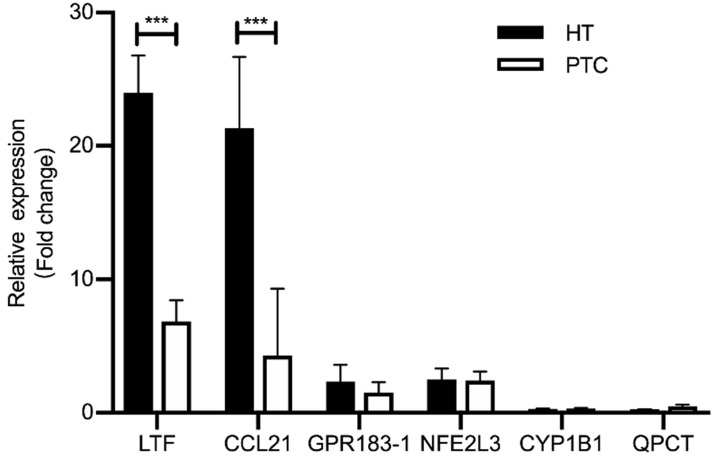
Expression of shared DEGs in HT and PTC samples compared with the control (n=5/group), as assessed by RT-PCR. *** P <0.001; * P <0.05.

We performed quantitative RT-PCR (qRT-PCR) using clinical samples to further verify the expression of shared DEGs. As illustrated in Fig. 6, compared with the PTC-adjacent normal tissues group, the HT group showed significantly increased mRNA levels of *LTF* and *CCL21* (P < 0.05) and the PTC group showed significantly decreased mRNA levels of *LTF* and *CCL21* (P < 0.05). These results were consistent with the findings of bioinformatics analysis.

## Discussion

Malignant events in the thyroid may be related to hypothyroidism and immune dysfunction, as chronic inflammation is thought to induce neoplastic transformation 3. Therefore, it is meaningful to assess associations between autoimmune thyroiditis and PTC; with subsequent development of tumor biomarkers as diagnostic and therapeutic targets. Prior studies have suggested that immune and inflammatory responses are significantly associated with HT and PTC occurrence.

In our study, some hub genes strongly related to the regulation of neoplastic transformation were identified among DEGs in HT. Mehta's group 33 used microarray analysis to detect that HLA class I deficiency frequently occurs in cervical carcinoma. Another study reported that HLA class I may have an important biological role as a mediator connecting immune-mediated recognition and tumors 34. With the help of HLA class I, cancer cells can escape identification and lysis by cytotoxic T lymphocytes as well as natural killer (NK) cell-mediated cytotoxicity 35, 36. Tyrosine kinase inhibitors (TKIs), which block tyrosine kinase activation, have been evaluated for the treatment of many kinds of cancer 37. Human PTC cell lines with rearrangements can be inhibited by pyrazolo[3,4-d] pyrimidine (PP), which is the most prominent inhibitor of LCK 38, 39. ITGB2 is a type of integrin that binds to intercellular adhesion molecule 1 on stromal cells, promoting cell adhesion to the extracellular matrix 40, 41. In a case-controlled study of *ITGB1* and *ITGB2* gene single-nucleotide polymorphisms (SNPs) in PTC patients, it was reported that *ITGB2* promoter polymorphisms are closely related to the development of PTC 42. Accordingly, there may be a connection between genetic mutations and autoimmune inflammatory diseases and cancer.

We employed RT-PCR to validate the importance of shared DEGs in clinical samples and found that mRNA expression levels of LTF and CCL21 were higher in HT tissues but lower in PTC tissues. LTF is an iron-binding protein related to the serum iron transport protein transferrin 43 that promotes B cell migration and maturation and the immune response, and B cells in turn enhances the antibody response by promoting the maturation of T cell precursors 44. Using indirect immunofluorescence, Afeltra's group identified anti-lactoferrin antibodies in 2 of 11 (18.1%) HT cases 45. In addition, LTF gene expression is decreased in thyroid cancer patients 46. These results are consistent with the findings of our study.

Infiltration of lymphocytes into the thyroid gland and the formation of lymph node-like structures are hallmarks of HT 47, and their occurrence correlates with expression of the chemokine CCL21 47, 48. CCL21 is a ligand of the chemokine receptor CCR7, activation of which promotes thyroid carcinoma growth 49. Indeed, CCL21 was significantly associated with lymphocyte infiltration in an RNA sequencing study using clinical samples from patients with T1N0 and T2-3N1 thyroid tumors carrying wildtype BRAF and the V600E mutation 50. In our study, *CCL21* was also more highly expressed in HT tissues than in PTC tissues.

Our study is the first to use bioinformatics analyses to identify and qPCR to verify shared DEGs related to HT and PTC. In addition, to identify connections between genetic mutations and HT and PTC, we analyzed some hub genes among HT-DEGs that are closely related to the regulation of neoplastic transformation. However, the main limitation of this research is that the microarray and qPCR analyses were based on gene and not protein expression; hence, the biomarkers identified in this study can only be utilized as gene markers. More studies should be performed to validate these DEGs in the future.

## Supplementary Material

Supplementary figure S1.

Supplementary tables.

## Figures and Tables

**Table 1:** GO-terms of co-expressed genes on AmiGO database

Gene	GO class (direct)	Evidence	Evidence	Reference
*LTF*	ossification	IEA	UniProtKB-KW:KW-0892	GO_REF:0000037
	regulation of cytokine production	IDA		PMID:20345905
	innate immune response in mucosa	IDA		PMID:12037568
	DNA binding	IEA	UniProtKB-KW:KW-0238	GO_REF:0000037
	iron ion binding	IDA		PMID:12037568
	protein binding	IPI	UniProtKB:P27958	PMID:12522210
	proteolysis	IEA	UniProtKB-KW:KW-0645	GO_REF:0000037
	negative regulation of ATPase activity	IMP		PMID:25193851
	negative regulation of apoptotic process	ISS	UniProtKB:P24627	GO_REF:0000024
	negative regulation of viral genome replication	IMP		PMID:25193851
	positive regulation of NF-kappaB transcription factor activity	IDA		PMID:20345905
	negative regulation of tumor necrosis factor (ligand) superfamily member 11 production	ISS		GO_REF:0000024
	negative regulation of osteoclast development	ISS		GO_REF:0000024
*CCL21*	positive regulation of dendritic cell antigen processing and presentation	ISS	UniProtKB:P84444	GO_REF:0000024
	protein binding	IPI	UniProtKB:Q86YL7	PMID:14978162
	immune response	TAS		PMID:10861057
	G protein-coupled receptor signaling pathway	IDA		PMID:18308860
	chemokine activity	ISS	UniProtKB:P84444	GO_REF:0000024
	positive regulation of glycoprotein biosynthetic process	ISS	UniProtKB:P84444	GO_REF:0000024
	positive regulation of receptor-mediated endocytosis	ISS	UniProtKB:P84444	GO_REF:0000024
	cell maturation	ISS	UniProtKB:P84444	GO_REF:0000024
	cellular response to chemokine	ISS	UniProtKB:P84444	GO_REF:0000024
	G protein-coupled receptor signaling pathway	IBA	UniProtKB:P13501	PMID:21873635
	cellular response to interleukin-1	IBA	UniProtKB:P13501	PMID:21873635
	cellular response to tumor necrosis factor	IBA	UniProtKB:P13501	PMID:21873635
	CCR chemokine receptor binding	IBA	UniProtKB:Q9Y258	PMID:21873635
	lymphocyte chemotaxis	IBA	UniProtKB:Q9Y258	PMID:21873635
	positive regulation of ERK1 and ERK2 cascade	IBA	UniProtKB:Q99731	PMID:21873635
	inflammatory response	IBA	UniProtKB:P78423	PMID:21873635

NOTES: ISS sequence similarity evidence used in manual assertion, IEA evidence used in automatic assertion, TAS traceable author statement used in manual assertion, IPI physical interaction evidence used in manual assertion, IBA biological aspect of ancestor evidence used in manual assertion, IDA direct assay evidence used in manual assertion.
