# Supplementary figures and images for: Bioinformatics analysis identified shared differentially expressed genes as potential biomarkers for Hashimoto's thyroiditis-related papillary thyroid cancer
Source: Int J Med Sci. 2021 Aug 13;18(15):3478–87. doi: 10.7150/ijms.63402 (PMC8436097; doi:10.7150/ijms.63402)

A

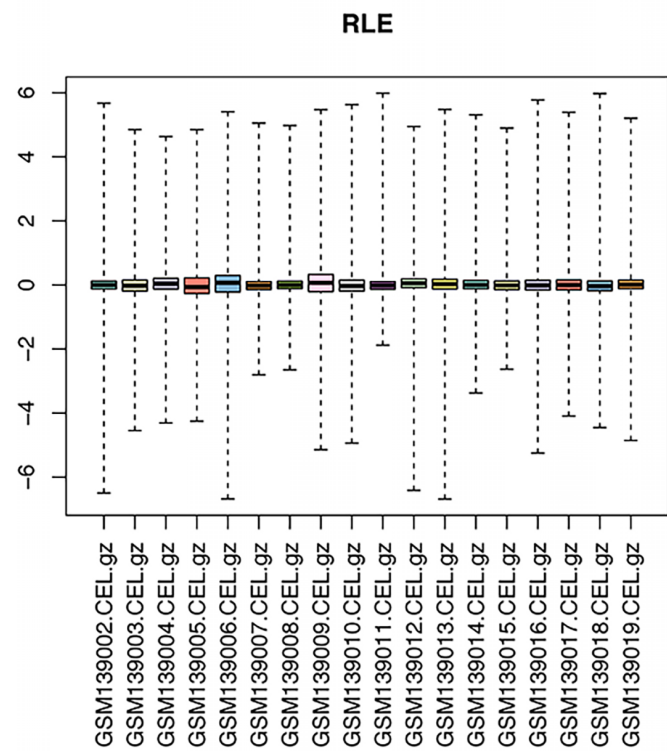

B

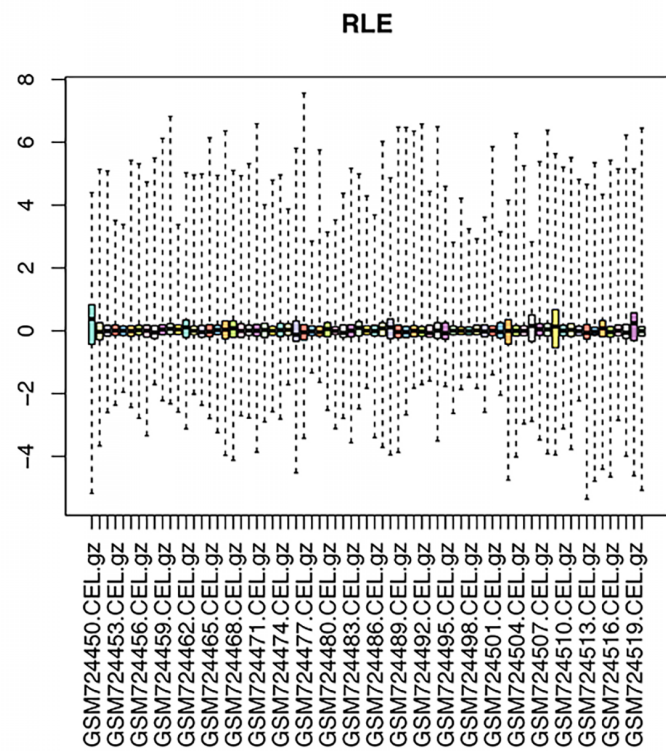

Supplement: Supplementary file 1 — Supplementary figure S1. [file ijmsv18p3478s1.pdf]
